# Myxoid Dermatofibrosarcoma Protuberans With Giant Cell Fibroblastoma Features in an Adult: Case Report and Review

**DOI:** 10.1177/10668969261438516

**Published:** 2026-05-07

**Authors:** Chenxu Shi, Ahmed Lazim, Paul J Zhang

**Affiliations:** 1 Department of Pathology and Laboratory Medicine, 21798 University of Pennsylvania Perelman School of Medicine, Philadelphia, PA, USA

**Keywords:** dermatofibrosarcoma protuberans (DFSP), myxoid subtype, giant cell fibroblastoma

## Abstract

Dermatofibrosarcoma protuberans (DFSP) is a rare, low-grade cutaneous sarcoma with a high local recurrence rate but low metastatic potential. The myxoid subtype of DFSP is exceptionally rare, and its occurrence with giant cell fibroblastoma features, particularly in adults, is even more uncommon, presenting diagnostic challenges. We describe a 32-year-old man with a myxoid DFSP exhibiting giant cell fibroblastoma features. The diagnosis was confirmed by immunohistochemistry and fluorescence *in situ* hybridization (FISH) for *PDGFB* rearrangement. This report highlights the diagnostic challenges in this rare entity and emphasizes the importance of molecular confirmation.

## Introduction

Dermatofibrosarcoma protuberans (DFSP) is a rare, locally aggressive cutaneous sarcoma that arises in the dermis and subcutaneous tissue. It is characterized by an infiltrative growth pattern of uniform spindle cells arranged in a storiform pattern and has a high rate of local recurrence but low metastatic potential. The pathogenesis of DFSP is strongly associated with a specific chromosomal translocation t(17;22)(q22;q13), resulting in the *COL1A1::PDGFB* fusion gene, which drives tumorigenesis by activating platelet-derived growth factor receptor β (PDGFRβ) signaling pathway.^1–3^

Among the histologic subtypes of DFSP, the myxoid subtype is particularly rare, representing less than 5% of tumors. Myxoid DFSP is distinguished by its abundant myxoid stroma, which can obscure the typical storiform growth pattern, making diagnosis more challenging. In rare instances, features reminiscent of giant cell fibroblastoma may be present in DFSP, including multinucleated giant cells and pseudovascular spaces.^1–4^ Giant cell fibroblastoma is considered a juvenile form of DFSP and typically occurs in children, although rare occurrences in adults have been reported.^4,5^ The presence of giant cell fibroblastoma features in myxoid DFSP in adults is exceedingly rare and raises diagnostic and therapeutic challenges.

## Case Report

A 32-year-old man presented with a slowly enlarging, protuberant mass on his left upper thigh/groin region. The lesion had been present for several months, causing mild irritation, especially during physical activity. The patient reported a history of a similar lesion in the same location, which excised 2 years prior, though no records or pathology report were available. Physical examination revealed a 3-cm firm, nodular mass with slight ulceration overlying the femoral vasculature. A preliminary clinical impression by the referring primary care physician suggested a keloid.

**Figure 1.**
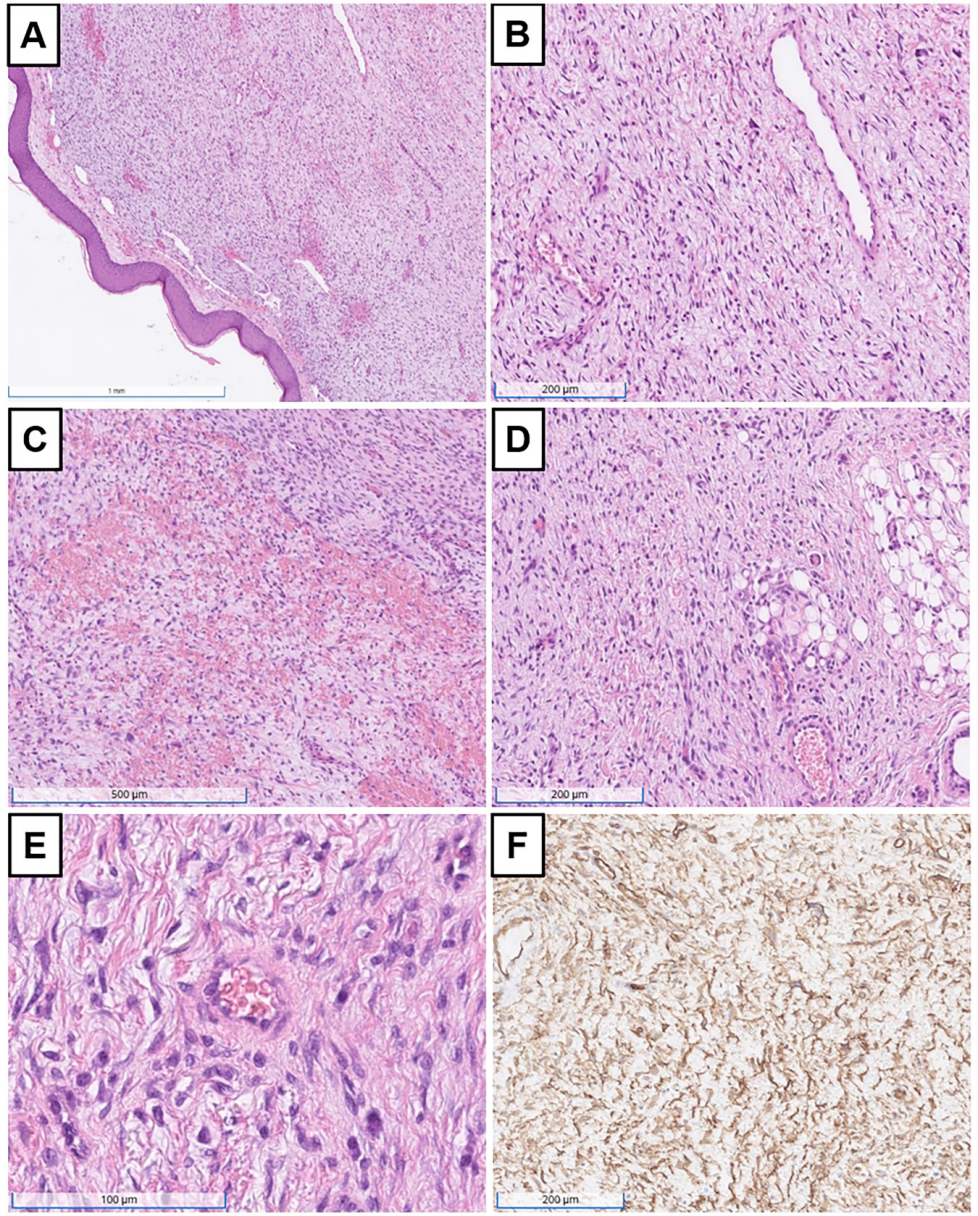
Histopathology of myxoid dermatoﬁbrosarcoma protuberans (DFSP) with giant cells. (A) DFSP with predominantly myxoid stroma. (B) Myxoid DFSP composed of elongated, uniform spindle cells and interspersed thin-walled vessels. (C) Transition from storiform fascicles of DFSP to a hypocellular myxoid area with multinucleated giant cells. (D) Giant cell fibroblastoma-like area showing adjacent myxoid DFSP with infiltration of subcutaneous adipose tissue in a honeycomb pattern. (E) Area containing bland spindle cells within fibrous and myxoid stroma, with pleomorphic and multinucleated giant cells lining pseudovascular clefts. (F) Diffuse CD34 reactivity in both mononucleated and multinucleated giant cells.

**Figure 2.**
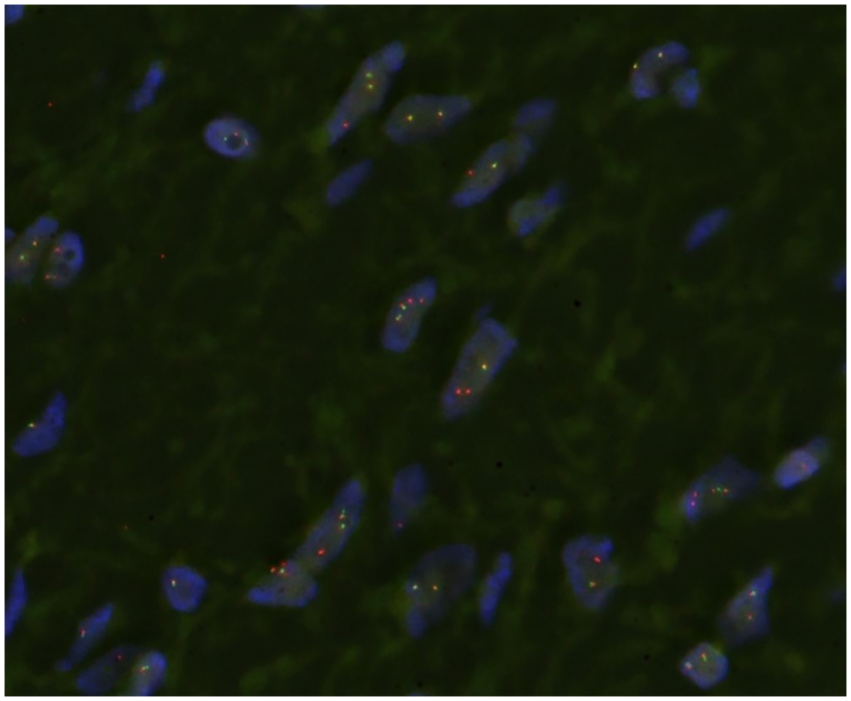
*COL1A1::PDGFB* rearrangement (fusion signal) detected by fluorescence *in situ* hybridization (FISH).

Excision was performed, and histopathological examination revealed a dermal-based, moderately cellular proliferation of bipolar and unipolar spindle and stellate cells with minimal nuclear atypia and scant eosinophilic cytoplasm. The stroma was predominantly myxoid, with prominent arborizing thin-walled vessels and scattered inflammatory cells, including neutrophils and eosinophils (Figure 1A-C). Small areas exhibited prominent storiform fascicles composed of uniform spindle cells with elongated thin nuclei, even chromatin, minimal cytoplasm, and indistinct cell borders, with transition to hypocellular myxoid areas containing multinucleated giant cells (Figure 1C). Adjacent areas demonstrated infiltration of subcutaneous adipose tissue in a honeycomb pattern, consistent with DFSP (Figure 1D). Additional areas showed multinucleated giant cells lining focal pseudovascular clefts within fibrous and myxoid stroma, supporting giant cell fibroblastoma features (Figure 1E). Rare mitotic figures were observed without evidence of necrosis, and no histologic features of fibrosarcomatous transformation were seen. Immunohistochemical staining showed diffuse positivity for CD34 (Figure 1F), while the tumor cells were negative for SMA, desmin, S100, SOX10, EMA, MUC4, and GLUT1 (*SLC2A1*). Fluorescence *in situ* hybridization (FISH) analysis revealed a *PDGFB* rearrangement (Figure 2), confirming the diagnosis of myxoid DFSP with giant cell fibroblastoma features.

Due to tumor involvement at inked specimen margins, the patient subsequently underwent a wide re-excision, achieving negative peripheral margins. However, the deep margin remained involved by the hypocellular component of the tumor, leading to sarcoma tumor board discussion. Imaging and an orthopedic oncology consultation were recommended to evaluate the possibility of a second re-resection. Given the low metastatic potential of DFSP, routine surveillance was advised. The patient remains under clinical surveillance, with no evidence of recurrence at 11 months following wide re-excision.

## Discussion

The myxoid DFSP poses diagnostic challenges due to its histologic overlap with other myxoid spindle cell neoplasms. Histologically, it must be distinguished from various benign and malignant cutaneous myxoid spindle cell neoplasms, including myxoid dermatofibroma, superficial angiomyxoma, myxoid solitary fibrous tumor (SFT), myxoid nerve sheath tumor, low-grade myxofibrosarcoma, and low-grade fibromyxoid sarcoma. Key distinguishing features of myxoid DFSP include its characteristic CD34 positivity, the presence of the *COL1A1::PDGFB* fusion gene, and the classic honeycomb infiltration of subcutaneous tissue.^1–3^^,6^ Immunohistochemical staining plays a critical role in differentiating these entities, with DFSP demonstrating strong, diffuse CD34 positivity, which is particularly helpful in distinction from myxoid dermatofibroma.^6^ Additional immunostains such as S100, Factor XIIIa, MUC4, STAT6, SMA, and desmin are also commonly used to help differentiate myxoid DFSP from other cutaneous myxoid tumors.

One of the primary entities in the differential diagnosis is myxoid dermatofibroma (fibrous histiocytoma), a benign dermal neoplasm that can display a prominent myxoid stroma, mimicking myxoid DFSP. However, myxoid dermatofibroma typically lacks the infiltrative growth pattern and storiform architecture seen in DFSP and is usually confined to the dermis without honeycomb entrapment of subcutaneous fat. Immunohistochemically, it often expresses Factor XIIIa but is usually negative for CD34. While both entities may share similar low-power appearance, dermatofibroma frequently exhibits peripheral collagen trapping and hemosiderin deposition, which are uncommon in DFSP.^7^

Superficial angiomyxoma is a benign cutaneous neoplasm characterized by a myxoid stroma with prominent vasculature and scattered inflammatory cells, particularly neutrophils. While it may show variable CD34 positivity, it lacks the storiform architecture and diffuse infiltrative growth pattern typical of DFSP. Additionally, the presence of neutrophils - a hallmark of superficial angiomyxoma- is uncommon in myxoid DFSP. In a subset of tumors, particularly those associated with Carney complex, superficial angiomyxoma demonstrates loss of PRKAR1A expression.^8^

Another consideration is myxoid SFT, which rarely involves the skin and displays alternating hypercellular and hypocellular areas with a hemangiopericytoma-like vascular pattern. Although myxoid SFT may express CD34, it is typically STAT6-positive, a feature absent in DFSP. Furthermore, myxoid SFT lacks the honeycomb infiltration pattern characteristic of DFSP, aiding in its distinction.^9^

Myxoid nerve sheath tumor, particular myxoid perineurioma is another diagnostically challenging entity, characterized by elongated spindle cells arranged in a storiform or fascicular pattern within a myxoid stroma. While myxoid perineurioma may show focal CD34 positivity, it is also positive for EMA and GLUT1, markers that are negative in DFSP. Myxoid schwannoma and neurofibroma may also show CD34 positivity, but their co-expression of S100 protein helps distinguish them from DFSP.^10,11^

Similarly, low-grade myxofibrosarcoma is an important differential diagnosis, as it exhibits an infiltrative growth pattern with irregular, curvilinear vasculature and pseudolipoblasts. Unlike DFSP, low-grade myxofibrosarcoma shows greater nuclear atypia and pleomorphism and lacks the characteristic *PDGFB* rearrangement, features that aid in differentiation.^12^

Low-grade fibromyxoid sarcoma is another potential mimic, often presenting as a circumscribed tumor with arcades of thick-walled blood vessels, a feature uncommon in myxoid DFSP. Immunohistochemically, the tumor expresses MUC4, and molecularly, it often harbors a *FUS* translocation, both of which are absent in DFSP.^13^

When diagnostic challenges arise due to morphologic overlap or vague immunophenotype, DFSP can be confirmed or distinguished by the detection of *PDGFB* rearrangement by FISH, which is negative in all the aforementioned cutaneous myxoid spindle cell tumors.

DFSP and giant cell fibroblastoma share a close histopathologic and molecular relationship, suggesting that they represent different morphologic expressions of the same neoplastic process. Both tumors are characterized by the *COL1A1::PDGFB* fusion gene, resulting from the t(17;22)(q22;q13) translocation.^14^ While DFSP predominantly affects adults and presents as an infiltrative spindle cell proliferation with a storiform pattern, giant cell fibroblastoma is more common in children and exhibits a myxoid stroma with pseudovascular spaces and multinucleated giant cells.^15^ Hybrid lesions containing both DFSP and giant cell fibroblastoma have been reported, and giant cell fibroblastoma can transition to DFSP upon recurrence, reinforcing the notion that these two entities exist on a histologic continuum.^4,5,16^ Importantly, despite age and morphologic differences, DFSP and giant cell fibroblastoma demonstrate similar clinical behavior, with a high rate of local recurrence when incompletely excised and a very low risk of metastasis, primarily in the setting of fibrosarcomatous transformation.^1,4,17^ When present, fibrosarcomatous transformation in DFSP is characterized by a high-grade spindle cell component with increased cellularity, nuclear atypia, and elevated mitotic activity, often accompanied by reduced or lost CD34 expression. This transformation is clinically significant, as it is associated with increased aggressiveness and metastatic potential, making its recognition critical for prognostication and management.^17^

The primary treatment for DFSP, including the myxoid subtype and tumors with giant cell fibroblastoma features, is wide surgical excision with negative margins. Mohs micrographic surgery can also be an effective option if performed correctly, particularly for tumors in anatomically challenging locations.^18^ Due to the high local recurrence rate, which can reach up to 50%, meticulous surgical planning and long-term follow-up are essential.^19^ For locally advanced or inoperable DFSP, tyrosine kinase inhibitors such as imatinib have shown efficacy, particularly in tumors harboring the *COL1A1::PDGFB* fusion gene.^20^ However, imatinib efficacy diminishes in tumors with fibrosarcomatous transformation. In such patients, anthracycline-based chemotherapy is typically recommended.^21,22^

This report underscores the diagnostic complexity of myxoid DFSP with giant cell fibroblastoma features, an entity that can closely mimic a variety of benign and malignant myxoid neoplasms. The integration of histopathologic assessment, immunohistochemical staining, and molecular studies remains critical for establishing an accurate diagnosis. Surgical resection with clear margins remains the gold standard for treatment, and long-term follow-up is warranted to monitor for recurrence.
